# Soil geochemistry and constraint of tree seedlings immediately after germination on *Macrotermes* termite mounds in the Kruger National Park, South Africa

**DOI:** 10.1002/ece3.11348

**Published:** 2024-05-16

**Authors:** Anthony J. Mills, Ruan van Mazijk, Jessica Laine Allen, Tercia Strydom

**Affiliations:** ^1^ Department of Soil Science Stellenbosch University Matieland South Africa; ^2^ C4 EcoSolutions (Pty) Ltd. Cape Town South Africa; ^3^ Department of Biological Sciences University of Cape Town Cape Town South Africa; ^4^ OneWorld Sustainable Investments Cape Town South Africa; ^5^ Scientific Services, South African National Parks Skukuza South Africa

**Keywords:** grass‐tree competition, herbaceous plants, savanna, soil nutrients, tree seedlings

## Abstract

*Macrotermes* termite mounds in the Kruger National Park occupy a significant part of the savanna landscapes, occurring at densities of up to 70 km^−2^ and often exceeding 10 m in width and 4 m in height. The mounds are usually devoid of trees, but have dense grass cover in wet years. As a result, these mounds form large patches of grassland amongst the wooded savanna. To our knowledge, it is not known why trees are largely excluded from the mounds. We analysed soil surface nutrient concentrations on and off mounds (0–2 cm deep, *n* = 80) to ascertain whether the availability of nutrients could be influencing competition between grasses and tree seedlings. The results showed that potential deficiencies in P, Ca, Cu, Zn and B in soils off the mounds are likely to be constraining plant growth. Notably, only B, with an average concentration of 0.19 mg kg^−1^, was likely to be limiting plant growth on the mounds. Notwithstanding likely interactions with herbivory and fire, we hypothesise that because grasses are far less susceptible to deficiencies of B than dicotyledonous trees, it is likely that grass competition with tree seedlings is considerably greater on mounds than off mounds.

## Graphical abstract

Many termite mounds in the Kruger National Park, South Africa, are devoid of trees amongst an otherwise wooded savanna, with no existing explanation for this vegetation pattern. We found potential deficiencies in multiple soil nutrients off the mounds that could constrain plant growth; of these nutrients, only boron was likely to be limiting on the mounds. We hypothesise that, because grasses are less susceptible to boron deficiency than dicotyledonous trees, grasses are likely to compete more with tree seedlings on than off termite mounds.

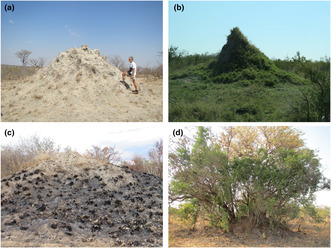

## 1 INTRODUCTION

Termites in savannas across the world are known to be ecosystem engineers because they alter soil physical and chemical properties, move large quantities of soil and increase rates of nutrient cycling. These effects on soils, which can take place over as much as 20% of savanna landscapes (Davies et al., 2016; Levick et al., 2010), in turn have a major effect on the distribution of plants and herbivores (Erpenbach et al., 2012; Moe et al., 2009; Muller & Ward, 2013; Muvengwi & Witkowski, 2020; Sileshi et al., 2010).

**FIGURE 1:**
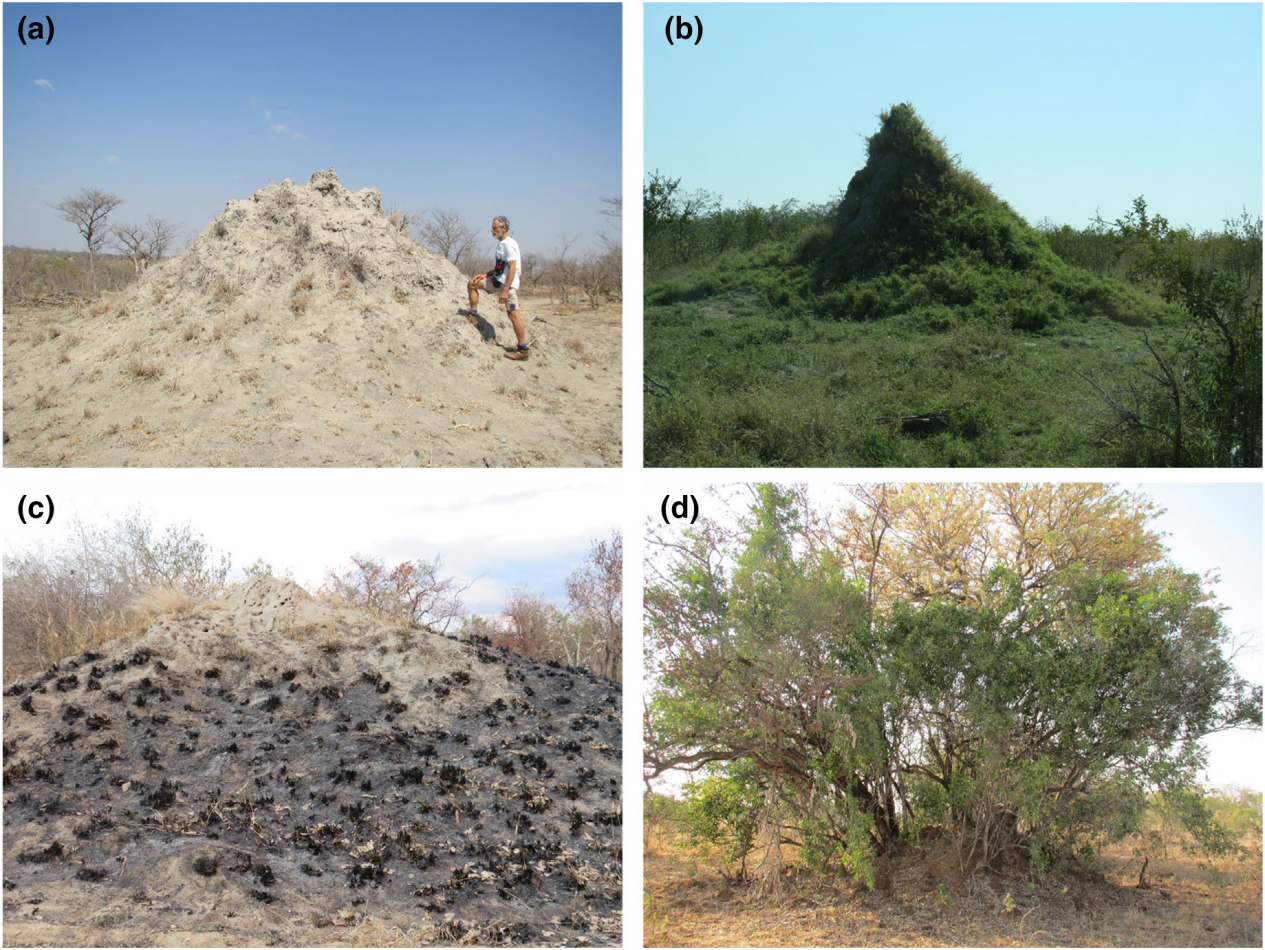
*Macrotermes* termite mounds near (a, d) Skukuza Rest Camp and (b, c) Phalaborwa in the Kruger National Park, South Africa. Pictured are (a) a mound in the dry season, with sparse and dormant grass bases, (b) a mound in the wet season, with a vigorous grass sward enveloping it, (c) a mound in the dry season after fire, with burnt, dormant grass bases and (d) a mound with trees growing out of it.

**FIGURE 2:**
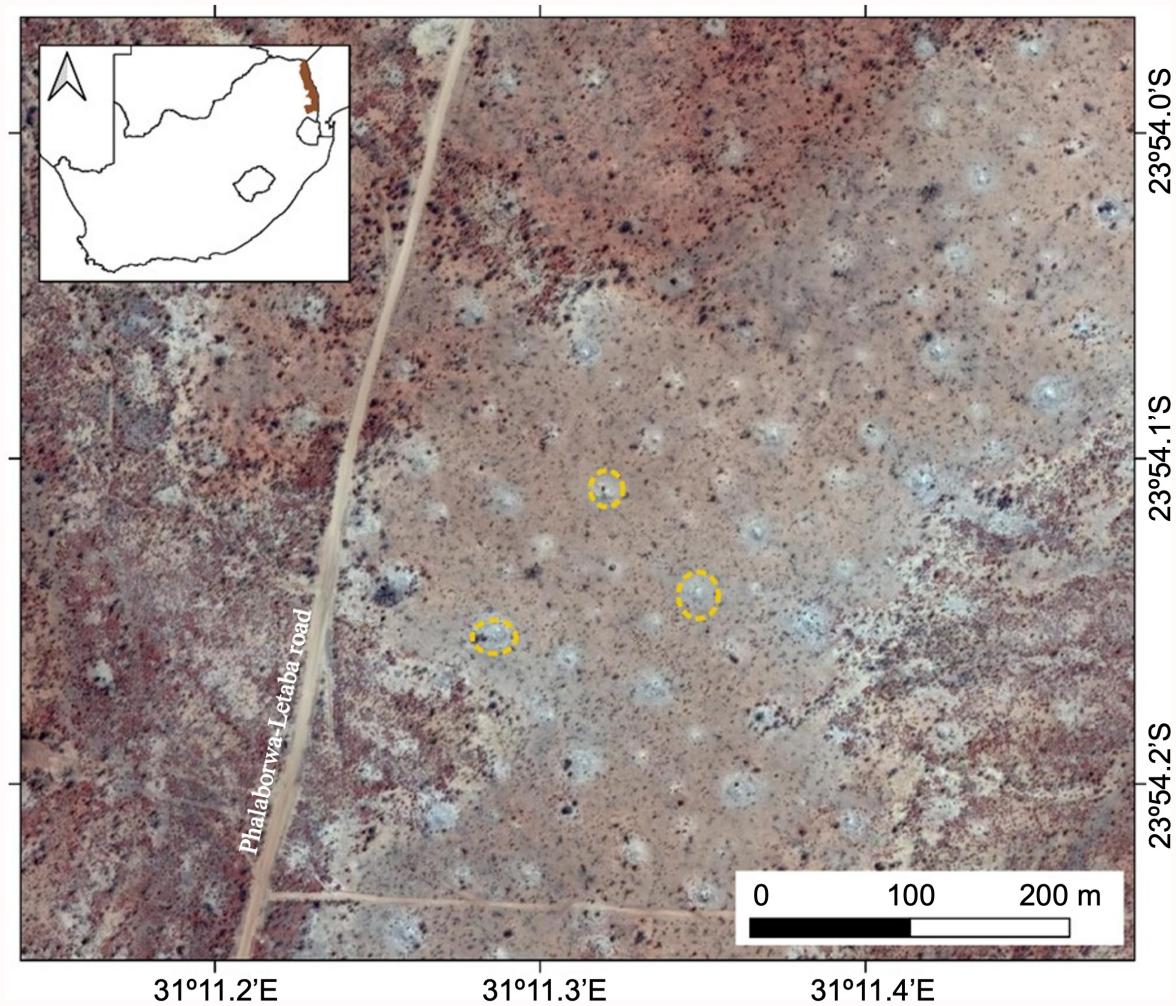
The distinct vegetation patterns produced by large *Macrotermes* termite mounds in the Kruger National Park. Three of the termite mounds have been circled in yellow. Imagery is from Google Earth (Map data © 2017 AfriGIS (Pty) Ltd.; Google Imagery © CNES/Airbus, DigitalGlobe, Landsat/Copernicus).

In many landscapes of the Kruger National Park, South Africa, *Macrotermes* termites form large mounds, which can occur at densities of up to 70 km^−2^ (Davies, Levick, et al., 2014) and can frequently exceed 10 m in diameter and 4 m in height (Figure 1). Despite the presence of many tree species in the savanna matrix between mounds, trees are often absent from these mounds (Figure 2), while grasses are abundant on them (Figure 1b) (Davies, Robertson, et al., 2014). Because the distance between mounds is frequently <100 m (Davies, Levick, et al., 2014), there is a substantial treeless grassland, albeit in discrete patches, formed by *Macrotermes* termites in these landscapes.

The factors leading to poor recruitment of tree seedlings (as opposed to other plants) on mounds relative to the savanna matrix have, to our knowledge, not been investigated. There are many potential factors warranting investigation, including damage by termites, herbivory (Mills et al., 2009), drought stress caused by a relatively high clay content, and competition from grasses (Morrison et al., 2019; Porensky & Veblen, 2012; Riginos, 2009; Tomlinson et al., 2019; Van Auken & Bush, 1988). The constraint of soil nutrients is likely to interact with termites, mammalian herbivory, drought and grass competition, given that sub‐optimal availability of any particular nutrient will adversely affect a recently germinated tree seedlings' rate of growth as well as recovery from damage (Cohn et al., 1989; Kambatuku et al., 2011; Vadigi & Ward, 2012). In this paper, we focus on identifying such potentially sub‐optimal nutrient concentrations in termite mounds and savanna soils. Our approach was to sample the soil surface of termite mounds and adjacent treed savannas as a first step in assessing the potential influence of nutrition on grass competition as well as tree seedling growth immediately after germination.

## 2 MATERIALS AND METHODS

Eighty composite samples (0–2 cm) were taken from treeless mounds and within adjacent savannas at a site ~20 km southwest of Skukuza Rest Camp (40 samples) and a site ~15 km northeast of Phalaborwa Gate in the Kruger National Park (40 samples) with permission from South African National Parks. Each composite sample comprised 8–10 sub‐samples taken from ~100 cm^2^ within a plot of ~100 m^2^. The samples in savannas were taken outside of tree canopies. The distance between a mound and its paired adjacent savanna sampling area was a maximum of 20 m. The Skukuza site is located within Granite Lowveld savanna, with Makhutswi Gneiss as the underlying geology (Mucina & Rutherford, 2006). The Phalaborwa site is located within Phalaborwa‐Timbavati Mopaneveld, with Nelspruit Granodiorite as the underlying geology (Mucina & Rutherford, 2006). We did not excavate soil pits and consequently did not classify the soils from our sites explicitly. However, a study by Venter (1990) across the KNP found that the most common soil forms at the Skukuza site were Hutton (on hillcrests), Longlands and Cartref (on midslopes) and Sterkspruit and Estcourt (on footslopes). The same study found that the most common soil forms at the Phalaborwa site were Clovelly and Glenrosa (on hillcrests), Cartref (on midslopes) and Estcourt, Shortlands and Hutton (on footslopes). Therefore, at both the Skukuza and Phalaborwa sites, the soils of the crests, midslopes and footslopes are noted to be similarly predominated by oxidic, lithic and duplex forms, respectively (Fey, 2010).

Soil samples were air‐dried and sieved to <2 mm. pH was determined using a 1:2.5 soil:1 M KCl solution. Inductively coupled plasma mass spectrometry was used to analyse: (i) K, Ca, Mg and Na (1% citric acid extract) (Division of Chemical Services, 1956); (ii) P (1% citric acid extract) (Du Plessis & Burger, 1965); (iii) S (calcium phosphate extract) (Beaton et al., 1968); (iv) B (hot water extract) (Bingham, 1982) and (v) Mn, Cu and Zn (0.02 M di‐ammonium ethylenediaminetetraacetic acid extract) (Beyers & Coetzer, 1971; Trierweiler & Lindsay, 1969). Organic C (SOC, %) was determined using the Walkley‐Black method (Nelson & Sommers, 1982; Walkley, 1935).

## 3 RESULTS AND DISCUSSION

**FIGURE 3:**
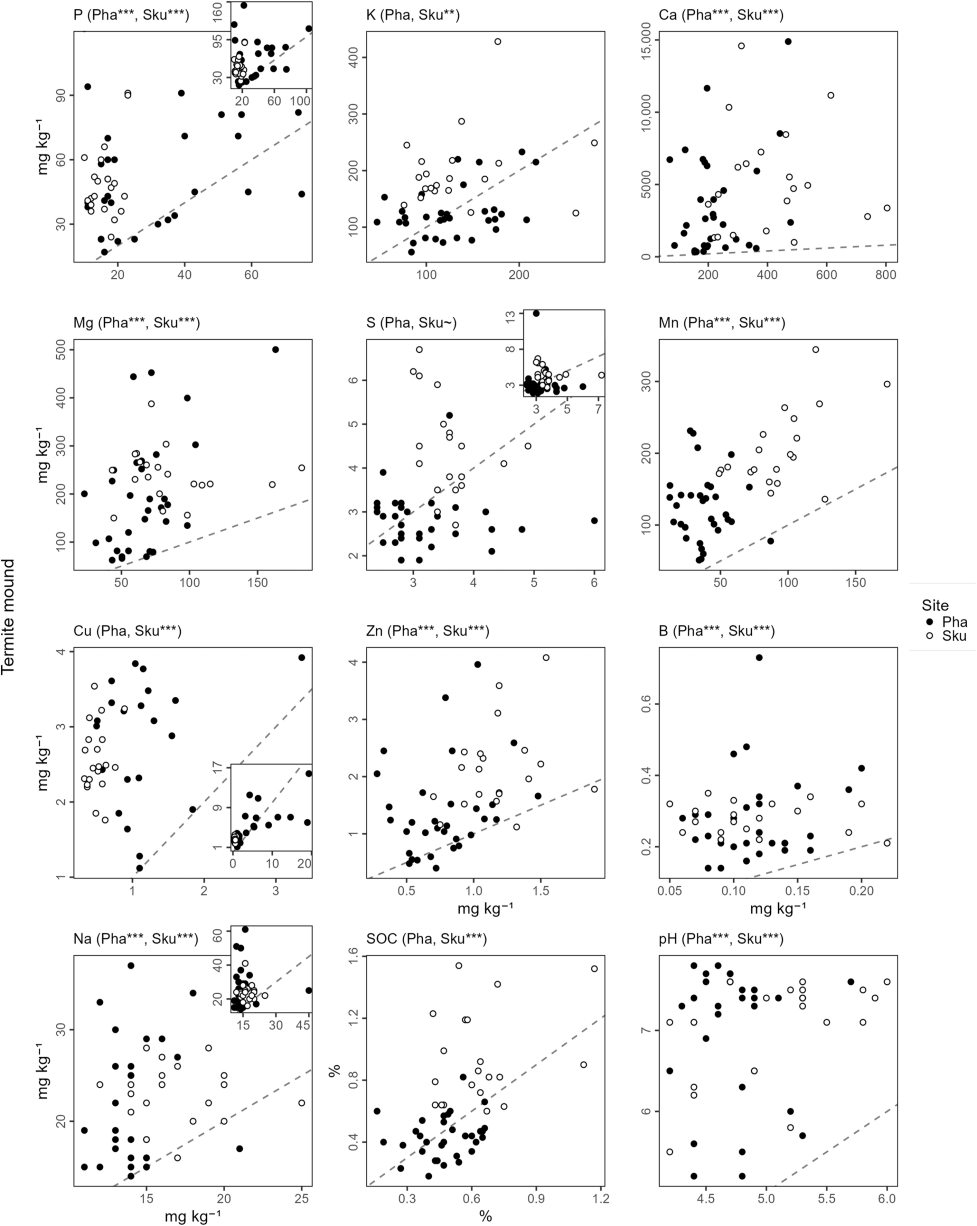
Soil chemistry of *Macrotermes* termite mounds versus adjacent savannas at the Skukuza (Sku) and Phalaborwa (Pha) sites in the Kruger National Park, South Africa (white and black points, respectively, as keyed). Insets in panels for P, S, Cu, Na present the full datasets for those elements, with the main panels cropped to the majority of the data (concentrations <100, 7, 4 and 40 mg kg^−1^, respectively). The need for such cropping is particularly notable for Cu, with some Phalaborwa mound and savanna soil samples having concentrations 5–20× higher than most samples (possibly due to the nearby copper‐vermiculite mine). Asterisks show significant differences between plots on termite mounds versus in adjacent savanna according to two‐sided paired *t*‐tests performed in R (R Core Team, 2022; Wickham et al., 2019) using the full datasets (***p < .001; **p < .01; *p < .05; ^~^ *p* < .1). The 1:1 lines are included for illustration.

The results (Figure 3) showed that, relative to the savanna, the soil surface of mounds was enriched at both sites in Ca, Mg, Mn, Cu, Zn, B and Na; was enriched at the Skukuza site in P and K and was more alkaline at both sites. The nutrient enrichment of the mounds relative to the savannas suggests that the competition from grasses that trees experience is likely to be more intense on mounds as a result of a greater availability of nutrients.

Of the nutrients that differed significantly between the savanna and mound soils (Figure 3), it is noteworthy that the concentrations of some of the nutrients analysed – namely P, Ca, Cu, Zn and B – are likely impeding plant growth at our study sites. All of these nutrients showed a positive correlation with the basal stem diameter of woody plants at the same study sites (Mills & Strydom, 2020). As these nutrients were found here at concentrations potentially limiting to plant growth (see below), this provides further support for the contention that these nutrients are likely to be constraining plant growth in the savannas under investigation. Assessing whether a given concentration of a soil nutrient from a specific soil will impede growth in a particular plant species is, however, complex, as multiple soil properties such as pH, clay mineralogy, soil water content and other nutrients' concentrations influence nutrient uptake by roots (Bell, 1997; Shorrocks, 1997; Wilkinson et al., 2000). Notwithstanding this complexity, on finding the concentrations of P, Ca, Cu, Zn and B shown in Figure 3 and assuming that the goal (at any given site, regardless of soil properties, climate, or plant species involved) was to optimise plant growth, it would be prudent to determine experimentally whether these nutrients are limiting plant growth. Below, we note the concentrations of these nutrients that have previously been found to limit plant growth in other studies.

Soils sampled away from the mounds at both savanna sites had concentrations of P averaging 26 mg kg^−1^, which is considerably less than concentrations that have been found to constrain a wide range of plants in many different soil types. Examples of studies showing such constraint include those by Holford and Cullis (1985a, 1985b), Holford et al. (1985), Holford and Crocker (1988) and Maier et al. (1989), which found that concentrations of 33, 49, 45 and 64 mg P kg^−1^, respectively, restricted the growth of the plants under investigation. While these authors used Bray II P, these values can be converted to 1% citric acid P with reasonable accuracy (× 0.87; *R* ^2^ = .72) (White et al., 2020); this conversion results in equivalent citric acid P concentrations of 29, 43, 39, 56 and 35 mg kg^−1^, respectively. As these limiting concentrations are greater than the average 26 mg kg^−1^, we found away from mounds at Skukuza and Phalaborwa, it is likely that the P concentrations at these sites are limiting plant growth.

With respect to Cu, the soils sampled away from the mounds at the Skukuza site averaged 0.5 mg kg^−1^, which is a concentration also likely to impede plant growth. Relevant studies in this regard include those by Reith (1968), Pizer et al. (1966) and Shukla and Behera (2019), which found that concentrations of 1.1, 1.6 and 0.6 mg Cu kg^−1^, respectively, restricted the growth of the plants under investigation or was at the lower extreme of the concentrations recorded in soils exhibiting sub‐optimal plant growth.

Concentrations of Ca and Zn in soils sampled away from mounds at the Phalaborwa site are also potentially limiting to plant growth. The average concentrations of Ca and Zn recorded in these samples were 227 and 0.8 mg kg^−1^, respectively. Studies relevant to understanding the potential effect of such concentrations on plant growth include those by Haby et al. (1990), Gupta and Mittal (1981) and Trierweiler and Lindsay (1969), which reported that concentrations of 250–500 mg Ca kg^−1^, 0.7 mg Zn kg^−1^ and 1.4 mg Zn kg^−1^, respectively, restricted the growth of the plants under investigation.

Lastly, the only nutrient that had average concentrations expected to limit plant growth in soils sampled both on and off mounds and at both sites was B, with an average concentration across all soils sampled of 0.19 mg kg^−1^ (0.27 and 0.12 mg kg^−1^ on and off the mounds, respectively). Studies of relevance include those by Li et al. (1978), Rashid et al. (1994), Rafique et al. (2002) and Yadav et al. (2017), which found that concentrations of 0.38, 0.50, 0.60 and 0.54 mg B kg^−1^, respectively, restricted the growth of the plants under investigation.

Given the complexity of interactions between nutrient availability, grass growth, tree seedling establishment, fire and herbivory, it is difficult to determine how vegetation structure on the termite mounds versus the surrounding savannas is influenced by the relative nutrient richness of the mound soils (with only B likely to be constraining plant growth) and the relative nutrient poverty of the surrounding savanna soils (with P, Ca, Cu, Zn and B likely to be constraining plant growth; see also Mills & Strydom, 2020). To stimulate further research into this complexity, we offer some hypotheses for consideration. First, grasses are likely to be more competitive than tree seedlings on the mounds because they are considerably less susceptible to B deficiency (Bell, 1997; Tariq & Mott, 2007) and can often outcompete tree seedlings in nutrient‐rich soils (Porensky & Veblen, 2012; Riginos, 2009; Van Auken & Bush, 1988). Second, the grass sward on mounds is likely to be considerably more nutritious than the grass sward off the mounds in the savannas, which would increase the herbivory pressure on the mounds. Such pressure is likely to constrain tree seedlings more than it would constrain the grass sward (Donaldson et al., 2022; Muvengwi et al., 2019; Voysey et al., 2021). Lastly, notwithstanding the greater herbivory pressure on termite mounds, relatively fast grass growth on the mounds in wetter years is likely to result in higher‐intensity fires on the mounds than off them (Figure 1c). More intense fires are also likely to favour grasses over tree seedlings. The net result is that the nutrient‐rich mounds at our study sites are likely to have far fewer tree seedlings establishing per unit area than in the more nutrient‐deficient savanna soils.

Although many of the termite mounds across the Kruger National Park tend to be grass‐dominated with minimal tree cover (like in Figures 1b and 2), there are some mounds with relatively large trees growing out of them (Figure 1d). To our knowledge, no explanation has been offered as to why trees are able to establish on some mounds but not others. Based on the findings of our study, we offer two explanations for further research. First, it is feasible that in some parts of the landscape the construction of termite mounds results in enrichment of B to the point that it constrains neither tree seedling nor grass growth. This could, for example, happen in low‐lying areas where B, which is a highly soluble nutrient, has concentrated through time (Argust, 1998; Berger, 1949; Kanwar & Singh, 1961). Second, it is possible that termites sometimes build their mounds around trees, giving the impression that the trees are growing out of the mounds.

Pot experiments with a wide range of savanna grass and tree species are likely to be of value for further investigation into the hypothesis that soil nutrition on termite mounds enhances the competitive strength of grasses more than that of recently germinated tree seedlings. The effects of P, Ca, Cu, Zn and B deficiencies on the competition between grasses and tree seedlings would be of particular relevance. In this regard, nutrient analyses of leaves from the pot experiments would be important in order to ascertain whether plants are deficient in a particular nutrient or not.

## AUTHOR CONTRIBUTIONS

**Anthony J. Mills:** Conceptualization (lead); data curation (equal); formal analysis (equal); funding acquisition (lead); investigation (lead); methodology (lead); writing – original draft (lead); writing – review and editing (supporting). **Ruan van Mazijk:** Data curation (supporting); formal analysis (equal); visualization (equal); writing – review and editing (equal). **Jessica Laine Allen:** Data curation (equal); formal analysis (equal); investigation (supporting); methodology (equal); visualization (equal). **Tercia Strydom:** Methodology (supporting); project administration (lead); resources (equal).

## FUNDING INFORMATION

We gratefully acknowledge the South African Department of Environmental Affairs, Natural Resources Management Programme and the National Research Foundation (grant number FA2005040700027) for funding this research.

## CONFLICT OF INTEREST STATEMENT

As part of their positions at C4 EcoSolutions (Pty) Ltd., RvM and JLA were under the employment of AJM during the course of this research. The remaining authors declare that this research was conducted in the absence of any commercial or financial relationships that could be construed as a potential conflict of interest.

### OPEN RESEARCH BADGES

This article has earned Open Data and Open Materials badges. Data and materials are available at https://figshare.com/s/f8f84c9f0c740fd4990e.

## Data Availability

The raw data and R‐code from this study are available at the Stellenbosch University institutional research data repository ‘SUNScholarData’ at https://doi.org/10.25413/sun.23715960.
